# Multiparametric ^18^F-FDG PET/MRI-Based Radiomics for Prediction of Pathological Complete Response to Neoadjuvant Chemotherapy in Breast Cancer

**DOI:** 10.3390/cancers14071727

**Published:** 2022-03-29

**Authors:** Lale Umutlu, Julian Kirchner, Nils-Martin Bruckmann, Janna Morawitz, Gerald Antoch, Saskia Ting, Ann-Kathrin Bittner, Oliver Hoffmann, Lena Häberle, Eugen Ruckhäberle, Onofrio Antonio Catalano, Michal Chodyla, Johannes Grueneisen, Harald H. Quick, Wolfgang P. Fendler, Christoph Rischpler, Ken Herrmann, Peter Gibbs, Katja Pinker

**Affiliations:** 1 Department of Diagnostic and Interventional Radiology and Neuroradiology, University Hospital Essen, University of Duisburg-Essen, 45147 Essen, Germany; lale.umutlu@uk-essen.de (L.U.); michal-kamil.chodyla@uk-essen.de (M.C.); johannes.grueneisen@uk-essen.de (J.G.); 2 Department of Nuclear Medicine, University of Duisburg-Essen and German Cancer Consortium (DKTK)-University Hospital Essen, 45147 Essen, Germany; wolfgang.fendler@uk-essen.de (W.P.F.); christoph.rischpler@uk-essen.de (C.R.); ken.herrmann@uk-essen.de (K.H.); 3 Department of Diagnostic and Interventional Radiology, University Dusseldorf, Medical Faculty, 40225 Dusseldorf, Germany; nils-martin.bruckmann@med.uni-duesseldorf.de (N.-M.B.); janna.morawitz@med.uni-duesseldorf.de (J.M.); antoch@med.uni-duesseldorf.de (G.A.); 4 Institute of Pathology, University Hospital Essen, West German Cancer Center, University Duisburg-Essen and the German Cancer Consortium (DKTK), 45147 Essen, Germany; saskia.ting@uk-essen.de; 5 Department Gynecology and Obstetrics, University Hospital Essen, University of Duisburg-Essen, 45147 Essen, Germany; ann-kathrin.bittner@uk-essen.de (A.-K.B.); oliver.hoffmann@uk-essen.de (O.H.); 6 Institute of Pathology, Medical Faculty, Heinrich-Heine-University and University Hospital Duesseldorf, 40225 Duesseldorf, Germany; lenajulia.haeberle@med.uni-duesseldorf.de; 7 Department of Gynecology, University Dusseldorf, Medical Faculty, 40225 Dusseldorf, Germany; eugen.ruckhaeberle@med.uni-duesseldorf.de; 8 Division of Abdominal Radiology, Massachusetts General Hospital, Boston, MA 02129, USA; onofriocatalano@yahoo.it; 9 Erwin L. Hahn Institute for Magnetic Resonance Imaging, University of Duisburg-Essen, 45141 Essen, Germany; harald.quick@uni-due.de; 10 High-Field and Hybrid MR Imaging, University Hospital Essen, University of Duisburg-Essen, 45147 Essen, Germany; 11 Department of Radiology, Memorial Sloan Kettering Cancer Center, New York, NY 10065, USA; gibbsp@mskcc.org (P.G.); pinkerdk@mskcc.org (K.P.)

**Keywords:** multiparametric ^18^F-FDG PET/MRI, radiomics, breast cancer, radiomics-based prediction of pathologic complete response

## Abstract

**Simple Summary:**

In breast cancer, the leading cancer type and the main cause of cancer death in women, achieving pathological complete response after neoadjuvant chemotherapy has been shown to be associated with prolonged overall survival. Hence, the correct assessment and the potential prediction of therapy response have recently become the focus of research. In this study, we predicted pathological complete response prior to neoadjuvant system therapy using ^18^F-FDG PET/MRI radiomics analysis of the breast. Hence, simultaneous ^18^F-FDG PET/MRI may enable a more individualized and targeted approach to treatment as well as pretherapeutic patient stratification.

**Abstract:**

Background: The aim of this study was to assess whether multiparametric ^18^F-FDG PET/MRI-based radiomics analysis is able to predict pathological complete response in breast cancer patients and hence potentially enhance pretherapeutic patient stratification. Methods: A total of 73 female patients (mean age 49 years; range 27–77 years) with newly diagnosed, therapy-naive breast cancer underwent simultaneous ^18^F-FDG PET/MRI and were included in this retrospective study. All PET/MRI datasets were imported to dedicated software (ITK-SNAP v. 3.6.0) for lesion annotation using a semi-automated method. Pretreatment biopsy specimens were used to determine tumor histology, tumor and nuclear grades, and immunohistochemical status. Histopathological results from surgical tumor specimens were used as the reference standard to distinguish between complete pathological response (pCR) and noncomplete pathological response. An elastic net was employed to select the most important radiomic features prior to model development. Sensitivity, specificity, positive predictive value, negative predictive value, and accuracy were calculated for each model. Results: The best results in terms of AUCs and NPV for predicting complete pathological response in the entire cohort were obtained by the combination of all MR sequences and PET (0.8 and 79.5%, respectively), and no significant differences from the other models were observed. In further subgroup analyses, combining all MR and PET data, the best AUC (0.94) for predicting complete pathologic response was obtained in the HR+/HER2− group. No difference between results with/without the inclusion of PET characteristics was observed in the TN/HER2+ group, each leading to an AUC of 0.92 for all MR and all MR + PET datasets. Conclusion: ^18^F-FDG PET/MRI enables comprehensive high-quality radiomics analysis for the prediction of pCR in breast cancer patients, especially in those with HR+/HER2− receptor status.

## 1. Introduction

Since neoadjuvant chemotherapy (NAC) was introduced as the first-line defense in the treatment of locally advanced breast cancer, its indications for administration have been gradually extended in pursuing pathological complete response (pCR), particularly in cancers with unfavorable tumor profiles [1,2]. While pCR has been shown to be associated with prolonged survival when compared to non-pCR (partial response or no response), less than 10–50% of breast cancer patients achieve pCR (depending on the intrinsic subtype). Hence, correct assessment of therapy response, and ultimately, the pretreatment prediction of therapy response, is highly desirable to facilitate personalized treatment and prevent delays in effective treatment for non-responders [3].

The introduction of radiomics as a method to convert imaging features into quantifiable data and their respective extraction has amplified the understanding of proteogenomics and its relation to cancer [4,5,6,7]. As the leading cancer type and the main cause of cancer death in women, breast cancer has been the focus of intensive research over the past years, leading to distinctive improvements in understanding breast cancer phenotyping and corresponding treatment [8,9,10]. In comparison, the prediction of treatment response to neoadjuvant chemotherapy based on imaging and radiomics instead of invasive tissue sampling is a fairly new research focus. This new method for predicting treatment response comes with two positive effects: first, its non-invasive nature decreases potential risks associated with invasive procedures, and second, it attends to the intratumoral heterogeneity of breast cancer by enabling whole-tumor analysis (in contrast to focal biopsy), an important factor that has gained reasonable attention in past years [11,12].

While the majority of studies on radiomics analysis are based on routine imaging methods such as CT or MRI, an increasing number of studies have implemented more elaborate imaging methods, such as multiparametric ^18^F-FDG PET/MRI, to facilitate an even more comprehensive imaging platform for feature extraction, with promising initial results [13,14].

Hence, the aim of this study was to assess whether the utilization of multiparametric ^18^F-FDG PET/MRI-based radiomics analysis is able to predict pCR in breast cancer patients prior to treatment and enhance pretherapeutic patient stratification by means of precision medicine.

## 2. Materials and Methods

### 2.1. Patients

In total, 73 patients were included in this study retrospectively. The local ethics committee approved this study, and due to the anonymization of data, written patient consent was waived. Inclusion criteria comprised newly diagnosed, biopsy-proven treatment-naïve breast cancer with (a) T2 or higher T-stage tumor, (b) triple-negative (TN) tumor of any size, or (c) tumor with a high-risk molecular profile (e.g., Ki67 > 14%, G3, or HER2neu overexpression). Datasets were excluded from radiomics analysis if they were incomplete. All included patients were part of a larger study investigating the utility of PET/MRI in the initial staging of women with newly diagnosed breast cancer. The same inclusion criteria as previously mentioned were applied here.

### 2.2. PET/MRI

All ^18^F-FDG PET/MRI examinations were performed on an integrated 3-Tesla PET/MRI system (Biograph mMR, Siemens Healthcare GmbH, Erlangen, Germany). All patients underwent a dedicated breast ^18^F-FDG PET/MR and a whole-body imaging scan [15], but only the PET/MR breast examinations were evaluated for this study. All patients fasted for six hours before a bodyweight-adapted dosage of ^18^F-FDG was injected (4 Mbq/kg bodyweight) and examinations were performed one hour after injection. A dedicated 16-channel breast radiofrequency (RF) coil (Rapid Biomedical, Rimpar, Germany) that was specifically designed and developed for use in integrated PET/MR imaging was used for breast examinations [16]. All patients were imaged in head-first prone position. The dedicated breast protocol comprised the following sequences:

(1) A transversal T2-weighted fat-saturated turbo-spin echo (TSE) with a slice thickness of 7 mm (TE 97 ms; TR 2840 ms; FOV 400 mm; phase FOV 75%; acquisition matrix 256 × 192, in-plane resolution 1.6 × 1.6 mm; TA 5:28 min);

(2) A transversal diffusion-weighted echo-planar imaging (EPI) sequence with a slice thickness of 5.0 mm (TR 8000 ms; TE 81 ms; b-values: 0, 400, and 800 s/mm^2^, matrix size 192 × 156; FOV 420 mm, phase FOV, 81.3%; GRAPPA, acceleration factor 2; in-plane resolution 2.2 × 2.2 mm; TA 2:34 min);

(3) Six repetitions of a transversal 3-dimensional fast low-angle shot T1w (FLASH) sequence with a slice thickness of 7 mm (TE 3.62 ms; TR 185 ms; FOV 400 mm; phase FOV 75%; acquisition matrix 320 × 240, in-plane resolution 1.3 × 1.3 mm) for dynamic contrast-enhanced imaging. 

After the first FLASH sequence, a dose of 2 mL/kg bodyweight gadoterate meglumine (Guerbet, Dotarem) was injected. Automated image subtraction was subsequently performed.

PET acquisition was performed in one bed position with an acquisition time of 20 min simultaneously with MRI data. PET image reconstruction was subsequently performed utilizing an iterative ordered-subset expectation–maximization algorithm, 3 iterations and 21 subsets, a Gaussian filter with 4 mm full width at half maximum, and a 256 × 256 image matrix for the breast and a 344 × 344 image matrix for the whole-body protocol. PET data were attenuation-corrected automatically using the implemented 4-compartment model attenuation map (μ-map) calculated from fat-only and water-only datasets, as obtained by Dixon-based sequences.

### 2.3. Image Analysis

^18^F-FDG PET/MRI data were evaluated by two board-certified radiologists with 14 and 5 years of experience in breast imaging and hybrid imaging, supported by a nuclear medicine physician with 15 years of experience. All images were imported into an open-source medical image viewer (Horos v.3.3.5) for image visualization and quantitative parameter extraction. Breast lesions were identified on post-contrast subtracted images, and lesion location was recorded.

### 2.4. Radiomics Analysis

All PET/MRI datasets were imported to dedicated software (ITK-SNAP v. 3.6.0) for lesion annotation, which was performed by a radiologist with 14 years of experience in breast imaging, on the subtracted second post-contrast time point using a semi-automated method. Cystic/necrotic areas and/or biopsy markers were excluded during annotation. 

Prior to radiomic feature calculations, all images were reduced to 32 gray levels, and all dynamic images were normalized to the pre-contrast phase, resulting in maps of percentage enhancement. For potential data class imbalances, adaptive synthetic sampling was applied to equalize class sizes [17]. A total of 101 radiomic features were calculated and grouped into six classes (22 first order, 26 based on gray-level co-occurrence matrices, 16 based on run-length matrices, 16 based on size zone matrices, 16 based on neighborhood gray-level dependence matrices, and 5 based on neighborhood gray-tone difference matrices) using CERR software [18].

### 2.5. Reference Standard 

Pretreatment biopsy specimens were used to determine tumor histology, tumor and nuclear grades, and immunohistochemical status, including estrogen receptor, progesterone receptor, and HER2. The proliferation index Ki-67 was recorded as <15% (low proliferation) or ≥15% (high proliferation) [19]. In the case of an equivocal HER2 status, lesions were additionally evaluated using fluorescence in situ hybridization and classified as positive if gene amplification was detected. Determination of HER2 status followed the ASCO/CAP 2018 guidelines [20].

According to current guideline recommendations, tumors were classified into luminal A, luminal B, HER2+-enriched, and triple-negative based on the immunohistochemical evaluation. Histopathological results from surgical tumor specimens were used as the reference standard to distinguish between complete pathological response (pCR) and noncomplete pathological response (non-pCR) [21]. Regression criteria by Sinn et al. were applied to assess therapy response, with a score of 4 considered to be pCR [22].

### 2.6. Statistical Analysis and Predictive Model Building

After the determination of the most important radiomic features by using an elastic net combining Lasso and ridge regression, a maximum of 6 features were selected for each model to avoid overfitting. With a limited dataset, it is inappropriate to select a large number of features. Utilizing support vector machines and 5-fold cross-validation, predictive models were developed in Matlab. The use of 6 features ensures that there are at least 5 cases per feature in the minority class (31 pathological complete responders) for the main analysis and at least 2 cases per feature in the sub-analyses. The data were analyzed in three groups, (1) entire cohort, (2) HR+/HER2− subgroup, and (3) TN/HER2+ subgroup [3], as standalone sequences/positron emission tomography (PET), apparent diffusion coefficient (ADC), T2, PET, dynamic phase 1, dynamic phase 2, dynamic phase 3, dynamic phase 4, and dynamic phase 5 and then in various combinations (all dynamic phases aggregated, all MR data aggregated, and all imaging data aggregated). Sensitivity, specificity, positive predictive value, negative predictive value, and accuracy were calculated for each model. 

## 3. Results

### 3.1. Patient Population and Breast Lesion Characteristics 

**Figure 1:**
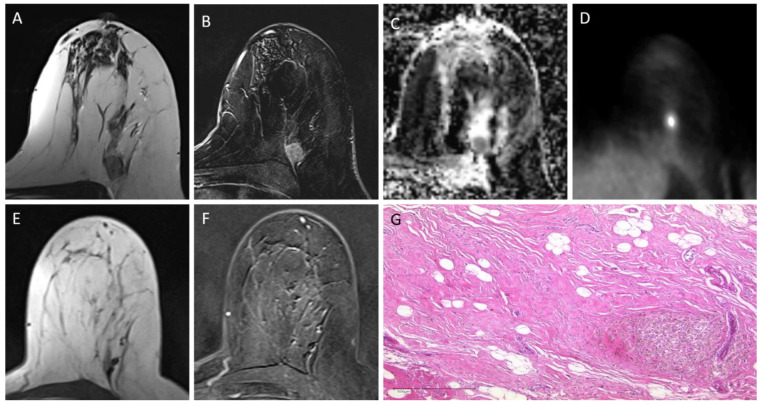
Example of a 47-year-old, triple-negative patient with pathological complete response after NACT: Primary tumor is clearly delineated in pretreatment breast MRI on T2w images (**A**) and post-contrast subtracted T1w images (**B**) and shows signal loss in the ADC map (**C**) and intense FDG uptake (**D**). In post-treatment breast MRI, no residual tumor can be detected on T2w images (**E**) or post-contrast subtracted T1w images (**F**,**G**). Post-therapeutic histopathology displaying focally accentuated sclerosing fibrosis and siderophages (lower right) but no residual invasive breast cancer (H&E, 50×).

The mean age of the 73 patients was 49 years (range 27–77 years). Of the 73 breast cancers, 47 were ER+ (64%), 48 were PR+ (66%), 21 were HER2+ (22%), and 69 showed high proliferation with Ki-67 greater than 15% (95%). Ten cancers were classified as luminal A (14%), forty-two were luminal B (58%), two were HER2-enriched (3%), and nineteen were triple-negative (TN) (26%). One cancer was classified as G1 (1%), 37 were G2 (51%), and 35 were G3 (48%). The cohort can be divided into 31 pathological complete responders (Figure 1) and 42 non-pathologic complete responders. Only four patients showed no reaction to neoadjuvant therapy (Sinn grade 0) and were included in the non-pCR group, as this population was too small for further subgroup analysis. In accordance with a publication by Braman et al., the cohort was further split for subgroup analyses into HR+/HER2− and TN/HER2+ cases. The HR+/HER2− group comprised 27 patients with non-pathological complete response and 14 with complete pathological response. In the TN/HER2+ subgroup, there were 15 patients with non-pathological complete response and 17 with complete pathological response. Please refer to Table 1 for detailed information on patient characteristics.

### 3.2. Prediction of Pathological Response in Entire Cohort

**Figure 2:**
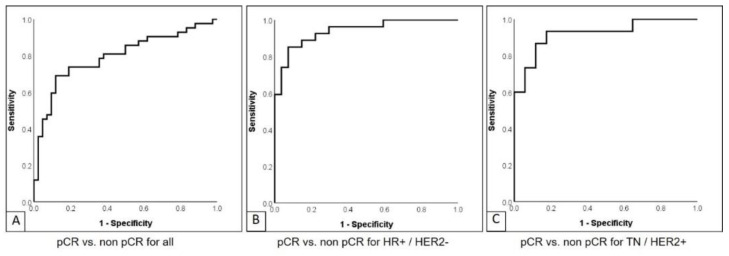
ROC for prediction of pathological response: ROC for prediction of pathological response based on the combination of all MR and PET data in (**A**) entire cohort, (**B**) in the subgroup of HR+/HER2− patients, and (**C**) in the subgroup of TN/HER2+ patients.

The best results in terms of AUC and NPV for the prediction of pCR were achieved by the combination of all MR and PET (0.8 and 79.5, respectively, see Figure 2A). Comparable AUC, sensitivity, and NPV were shown for PET only, resulting in an AUC of 0.77, sensitivity of 81%, and NPV of 78.9%. No significant differences among the results were observed. The lowest AUCs were reported for the second dynamic set (dynamic 2; 0.66), followed by the first dynamic set (0.69), all dynamics (0.69), and T2-weighted imaging (0.7). Please refer to Table 2 for detailed information on the best classification accuracies for the prediction of pCR and Table S1 in the Supplementary Material for detailed information on the selected features.

### 3.3. Subgroup Analysis 1: Prediction of pCR in HR+/HER2−

The best results in terms of the highest AUC for the prediction of pCR in HR+/HER2− patients were achieved by the combination of all MR and PET (0.94, see Figure 2B), followed by PET only (0.9) and all dynamics and all MR (both 0.89). While the highest sensitivity was shown for all dynamics (92.6%), the highest specificity was seen in T2-weighted (T2w) imaging (92.6%). T2w imaging also achieved the highest PPV (90.0%), while the best NPV was shown to be equal for PET and all MR and PET (85.2%). Please refer to Table 3 for detailed information on the best classification accuracies for the prediction of pCR and Table S2 in the Supplementary Material for detailed information on the selected features.

### 3.4. Subgroup Analysis 2: Prediction of pCR in TN/HER2+

The best results in terms of the highest AUC, sensitivity, specificity, PPV, NPV, and accuracy for the prediction of therapy response in TN/HER2+ patients were equally achieved by the combination of all MR and PET (see Figure 2C), all MR, and all dynamics (0.92, 88.2%, 86.7%, 88.2%, 86.7%, and 87.5%, respectively). The overall results for PET were only distinctly lower in this subgroup when compared to patients with HR+/HER2− as well as the entire cohort, with an AUC of 0.67, sensitivity of 70.6%, specificity of 60.0%, PPV of 66.7%, NPV of 64.3%, and accuracy of 65.6%. Comparably low results were obtained for the fifth dynamic set and ADC. Please refer to Table 4 for detailed information on the best classification accuracies for the prediction of pCR and Table S3 in the Supplementary Material for detailed information on the selected features.

## 4. Discussion

Radiomics-based analysis of breast cancer has emerged to become a well-investigated research focus in assessing its potential for predicting various endpoints, such as relapse, progression-free survival, subtype, or tumor phenotyping [7,8,9,10,13,14,23,24,25]. PCR after NAC has been shown to imply prolonged disease-free and overall survival [26] and has therefore been proposed as a surrogate early clinical endpoint for long-term survival [27]. Hence, the prediction of pCR to NAC has recently become the focus of radiomics-based research, supporting the idea of enhanced personalized medicine by means of pretherapeutic patient stratification. Whilst most studies showed promising results, the majority of them were based either on mammographic or MR-based imaging, so they did not involve the assessment of metabolic tumor features [6,10,28,29,30,31]. In this study, we aimed to assess a more comprehensive imaging platform that comprises morphologic, functional, and metabolic tumor features by means of simultaneous ^18^F-FDG PET/MR imaging for radiomics-based algorithmic prediction of pCR to NAC in patients with breast cancer. Our results are in line with previous studies demonstrating the general feasibility of MRI-based radiomics prediction of pCR to NAC and furthermore underline the added value of metabolic features, as the combined analysis of morphologic, functional, and metabolic tumor features achieved the best results in the entire cohort as well as in the subgroup with HR+/HER2. One of the early investigations on MRI-based radiomics prediction of pCR to NAC was published by Braman et al. [3]. Their results ranged from an AUC of 0.78 and accuracy of 0.76 in the training set to an AUC of 0.74 and accuracy of 0.67 in the testing dataset. Despite the distinct difference in the radiomic analysis performed by Bramann et al. in terms of their addition of peritumoral radiomics compared to our more limited intratumoral analysis, our results are comparable to theirs, yielding an AUC of 0.76 and accuracy of 0.70 based on all MR sequences. These results were further improved in our study once the PET component was added to the analysis (AUC 0.8; accuracy 77.4%), which underlines the reflection of metabolic features in tumor lesions and the added value for the prediction of pCR. Comparable to the results of Braman et al., our receptor-specific subgroup analyses also revealed better results than in the entire patient cohort. The prediction of pCR in patients with HR+, HER2− tumors achieved an AUC of 0.89 and accuracy of 83.3% based on all MR sequences and showed better results after the addition of PET (0.94 and 85.3%, respectively). Again, our results showed an improved tendency when compared to Braman et al. for the TN/HER2+ subgroup (AUC 0.92 and accuracy 87.5% in our study versus 0.89 and 83.3%) based on all MR sequences. The distinct difference between the TN/HER2+ subgroup and the results of the entire cohort and HR+/HER2− tumors was that PET did not add any valuable information in the TN/HER2+ group; hence, the results for all MR sequences and all MR and PET are identical. It is worth noting the differences in accuracy when considering PET-based radiomic features between the main analysis (76.2%) and the subgroup analyses (65.6% for TN/HER2+ cases and 87.5% for TN/HER2− cases). With a limited dataset (and thus large confidence intervals for diagnostic metrics), it is difficult to draw definitive conclusions, but these results seem to indicate that it may be appropriate to develop distinct models for predicting response based on subtype.

Examining the selected features in detail (Table S1), it is apparent that there is value in incorporating radiomic features from both modalities and a range of MR sequences (DWI, DCE, and T2) when developing a predictive model for the entire cohort. Interestingly, when the cohort is split into two subgroups, only the radiomic features from the DCE data are utilized in the final model for the TN/HER2+ cases. Conversely, radiomic features from PET imaging appear to predominate when analyzing HR+/HER2− cases. These observations warrant further investigation in a larger patient cohort.

The value of metabolic tumor features for the prediction of pCR has been previously demonstrated in a number of studies. Cheng et al. evaluated the utility of textural features of ^18^F-FDG PET/CT for predicting pCR after two cycles of chemotherapy. According to their results, the analysis of imaging parameters such as maximum standardized uptake value, metabolic tumor volume, total lesion glycolysis, and textural features, including entropy, coarseness, and skewness, enables the prediction of pCR in both HER2-negative and HER2-positive patients [32]. The predictive efficacy of PET/CT was further underlined in a more recent study by Li et al., as they could demonstrate that radiomic predictors from pretreatment PET/CT scans were able to predict pCR after NAC with accuracies of up to 0.8 (when combined with patient age) [33]. While the general feasibility and efficacy of PET/CT for the prediction of pCR in breast cancer has been well demonstrated, the utilization of PET/MRI as the imaging platform has been rather scarce. The recent publication by Choi et al. is among the few that used retrospectively fused PET/CT and MRI datasets to investigate their predictive efficacy for the prediction of pCR and compare an image deep learning model (CNN) with conventional methods. Their results revealed that the application of the CNN method further improved the accuracy of prediction compared to the conventional analysis in a subgroup of patients [34]. While their results can be considered promising, the highly selective and small patient cohort of 56 patients with a focus on TN- or HER2-negative cancers and rather low response rates to NAC (89% non-responders) limit the generalization of their results to the whole breast cancer population. 

To the best of our knowledge, our study is one of the first to utilize simultaneous ^18^F-FDG PET/MRI as the imaging platform for radiomic prediction of pCR to NAC. Our setup to analyze each MRI sequence and PET individually as well as in combination helped to gain more insight into the predictive efficacy of multiparametric imaging. While MRI sequences by themselves (ADC, T2, and DCE) showed rather poor predictive potential, the combined analysis of all MR sequences provided valuable AUC and accuracy values and was further improved after the addition of PET (except in the TN/HER2+ subgroup). This supports our hypothesis that the utilization of multiparametric ^18^F-FDG-PET/MRI may provide more comprehensive insight into breast cancer characteristics and hence serve as a valuable platform for the non-invasive prediction of pCR to NAC in breast cancer patients.

Although our results are promising regarding the potential of ^18^F-FDG PET/MRI as a platform for the radiomics-based prediction of pCR to NAC, the following important limitations of the current study should be noted: Ideally, feature selection should be performed within each fold to ensure full independence for the cross-validation analysis. However, with a limited dataset, the described approach was taken. This has the advantage of producing individual models for each sequence-type approach, rather than potentially multiple models reflecting variations in feature selection within each fold. The models and features described in this work can easily be applied to future datasets, enabling independent assessment of model accuracies. Previous publications demonstrated the benefits of including clinical features in imaging radiomic features for analysis [7] as well as in multi-center studies to assess the real value and clinical applicability of radiomics analyses. While the utilization of simultaneous PET/MR scanners is highly convenient, the rather low availability of integrated PET/MR scanners may hinder their widespread application. Hence, as shown in previous publications, the utilization of co-registered PET/CT and breast MRI data may accelerate the universal application of this valuable imaging platform for radiomics analysis. Overall, the past few years have demonstrated the promising value of the utilization of radiomics analyses in medicine. Nevertheless, it is important to acknowledge its current status as a research innovation, where the transition to clinical application is yet to be evaluated and implemented. These aspects should be addressed in future multi-center studies.

## 5. Conclusions

Overall, our results demonstrate that the combined analysis of metabolic, functional, and morphologic features facilitates a comprehensive platform for the accurate, non-invasive prediction of pCR to NAC in breast cancer patients. Hence, simultaneous ^18^F-FDG PET/MRI may help to develop a more individualized and targeted approach to treatment as well as pretherapeutic patient stratification.

## Figures and Tables

**Table 1:** Patient characteristics.

Total Patients		Number of Patients
Menopause status		
	Pre	38
	Peri	4
	Post	31
Ki67		
	Negative < 15%	4
	Positive > 15%	69
PR status		
	Positive	48
	Negative	25
ER status		
	Positive	47
	Negative	26
HER2neu expression		
	0	29
	1+	22
	2+	6
	3+	16
Tumor grade		
	G1	1
	G2	37
	G3	35
Subtype		
	Basal-like/ triple-negative	19
	Luminal A	10
	Luminal B	42
	Her2-enriched	2
Histology		
	NST	69
	Lobular invasive	3
	Other	1
Response (Regression criteria of Sinn)		
	0	4
	1	31
	2	4
	3	3
	4	31

**Table 2:** Best mean classification accuracies achieved for the prediction of pCR based on each MRI imaging sequence as well as in combination with PET for prediction of pCR in the entire cohort.

h	AUC (95% CI)	Sensitivity (95% CI)	Specificity (95% CI)	PPV (95% CI)	NPV (95% CI)	Accuracy (95% CI)
ADC	0.72 (0.61–0.83)	71.4 (55.4–84.3)	69.0 (52.9–82.4)	69.8 (58.6–79.0)	70.7 (59.0–80.3)	70.2 (59.3–79.7)
T2	0.70 (0.58–0.82)	76.2 (60.6–88.0)	69.0 (52.9–82.4)	71.1 (60.3–80.0)	74.4 (61.9–83.8)	72.6 (61.8–81.8)
Dynamic 1	0.69 (0.58–0.81)	66.7 (50.5–80.4)	69.0 (52.9–82.4)	68.3 (56.6–78.0)	67.4 (56.3–76.9)	67.9 (56.8–77.6)
Dynamic 2	0.66 (0.54–0.78)	71.4 (55.4–84.3)	59.5 (43.3–74.4)	63.8 (53.9–72.7)	67.6 (54.9–78.1)	65.5 (54.3–75.5)
Dynamic 3	0.71 (0.60–0.82)	47.6 (32.0–64.6)	78.6 (63.2–89.7)	69.0 (53.5–81.1)	60.0 (51.9–67.6)	63.1 (51.9–73.4)
Dynamic 4	0.71 (0.60–0.82)	38.1 (23.6–54.4)	92.9 (80.5–98.5)	84.2 (62.7–94.4)	60.0 (53.8–65.9)	65.5 (54.3–75.5)
Dynamic 5	0.72 (0.61–0.84)	69.0 (52.9–82.4)	71.4 55.4–84.3)	70.7 (59.0–80.3)	69.8 (58.6–79.0)	70.2 (59.3–79.7)
All Dynamics	0.69 (0.57–0.81)	66.7 (50.5–80.4)	76.2 (60.6–88.0)	73.7 (61.0–83.4)	69.6 (59.1–78.4)	71.4 (60.5–80.8)
All MR	0.76 (0.66–0.86)	71.4 (55.4–84.3)	69.0 (52.9–82.4)	69.8 (58.6–79.0)	70.7 (59.0–80.3)	70.2 (59.3–79.7)
PET	0.77 (0.66–0.87)	81.0 (65.9–91.4)	71.4 (55.4–84.3)	73.9 (63.2–82.4)	78.9 (66.1–87.8)	76.2 (65.7–84.8)
All MR and PET	0.80 (0.70–0.89)	81.0 (65.9–91.4)	73.8 (58.0–86.1)	75.6 (64.6–84.0)	79.5 (66.9–88.1)	77.4 (67.0–85.8)

**Table 3:** Best mean classification accuracies achieved for prediction of pCR based on each MRI imaging sequence as well as in combination with PET for prediction of pCR in HR+/HER2−.

Images	AUC	Sensitivity	Specificity	PPV	NPV	Accuracy
ADC	0.64 (0.48–0.79)	70.4 (49.8–86.3)	63.0 (42.4–80.6)	65.5 (52.3–76.7)	68.0 (52.6–80.3)	66.7 (52.5–78.9)
T2	0.85 (0.74–0.95)	66.7 (46.0–83.5)	92.6 (75.7–99.1)	90.0 (69.8–97.2)	73.5 (61.7–82.7)	79.6 (66.5–89.4)
Dynamic 1	0.79 (0.67–0.91)	70.4 (49.8–86.3)	74.1 (53.7–88.9)	73.1 (57.8–84.3)	71.4 (57.3–82.3)	72.2 (58.4–83.5)
Dynamic 2	0.66 (0.51–0.81)	59.3 (38.8–77.6)	70.4 (49.8–86.3)	66.7 (50.8–79.5)	63.3 (50.8–74.3)	64.8 (50.6–77.3)
Dynamic 3	0.69 (0.54–0.84)	85.2 (66.3–95.8)	59.3 (38.8–77.6)	67.6 (56.4–77.2)	80.0 (60.6–91.2)	72.2 (58.4–83.5)
Dynamic 4	0.72 (0.58–0.86)	66.7 (46.0–83.5)	77.8 (57.7–91.4)	75.0 (58.5–86.5)	70.0 (56.9–80.5)	72.2 (58.4–83.5)
Dynamic 5	0.65 (0.50–0.81)	81.5 (61.9–93.7)	63.0 (42.4–80.6)	68.8 (56.6–78.8)	77.3 (59.4–88.8)	72.2 (58.4–83.5)
All Dynamics	0.89 (0.80–0.98)	92.6 (75.7–99.1)	74.1 (53.7–88.9)	78.1 (65.2–87.2)	90.9 (72.1–97.5)	83.3 (70.7–92.1)
All MR	0.89 (0.79–0.98)	81.5 (61.9–93.7)	85.2 (66.3–95.8)	84.6 (68.6–93.3)	82.1 (67.3–91.2)	83.3 (70.7–92.1)
PET	0.90 (0.81–0.98)	85.2 (66.3–95.8)	85.2 (66.3–95.8)	85.2 (69.7–93.5)	85.2 (69.7–93.5)	85.2 (72.9–93.4)
All MR and PET	0.94 (0.88–1.00)	85.2 (66.3–95.8)	85.2 (66.3–95.8)	85.2 (69.7–93.5)	85.2 (69.7–93.5)	85.2 (72.9–93.4)

**Table 4:** Best mean classification accuracies achieved for prediction of pCR based on each MRI imaging sequence as well as in combination with PET for prediction of pCR in TN/HER2+.

Images	AUC	Sensitivity	Specificity	PPV	NPV	Accuracy
ADC	0.64 (0.43–0.85)	82.4 (56.6–96.2)	60.0 (32.3–83.7)	70.0 (54.7–81.8)	75.0 (49.8–90.1)	71.9 (53.5–86.3)
T2	0.75 (0.57–0.93)	76.5 (50.1–93.2)	66.7 (38.4–88.2)	72.2 (54.8–84.8)	71.4 (49.7–86.4)	71.9 (53.3–86.3)
Dynamic 1	0.75 (0.57–0.93	82.4 (56.6–96.2)	46.7 (21.3–73.4)	63.6 (50.9–74.7)	70.0 (42.2–88.2)	65.6 (46.8–81.4)
Dynamic 2	0.74 (0.55–0.93)	82.4 (56.6–96.2)	73.3 (44.9–92.2)	77.8 (59.5–89.3)	78.6 (55.7–91.5)	78.1 (60.0–90.7)
Dynamic 3	0.82 (0.65–0.98)	88.2 (63.6–98.5)	80.0 (51.9–95.7)	83.3 (64.2–93.3)	85.7 (61.4–95.8)	84.4 (67.2–94.7)
Dynamic 4	0.83 (0.68–0.98)	82.4 (56.6–92.4)	73.3 (44.9–92.2)	77.8 (59.5–89.3)	78.6 (55.7–91.5)	78.1 (60.0–90.7)
Dynamic 5	0.59 (0.38–0.80)	64.7 (33.3–85.8)	66.7 (38.4–88.2)	68.8 (49.8–83.0)	62.5 (44.4–77.7)	65.6 (46.8–81.4)
All Dynamics	0.92 (0.82–1.00)	88.2 (63.6–98.5)	86.7 (59.5–98.3)	88.2 (67.1–96.5)	86.7 (63.5–96.0)	87.5 (71.0–96.5)
All MR	0.92 (0.82–1.00)	88.2 (63.6–98.5)	86.7 (59.5–98.3)	88.2 (67.1–96.5)	86.7 (63.5–96.0)	87.5 (71.0–96.5)
PET	0.67 (0.48–0.86)	70.6 (44.0–89.7)	60.0 (32.3–83.7)	66.7 (50.0–80.0)	64.3 (43.6–80.7)	65.6 (46.8–81.4)
All MR and PET	0.92 (0.82–1.00)	88.2 (63.6–98.5)	86.7 (59.5–98.3)	88.2 (67.1–96.5)	86.7 (63.5–96.0)	87.5 (71.0–96.5)

## Data Availability

The data presented in this study are available on request from the corresponding author.

## Supplementary Materials

The following supporting information can be downloaded at: https://www.mdpi.com/article/10.3390/cancers14071727/s1, Table S1: Selected features (in order of importance) by elastic net regularization for each dataset for assessment of the entire cohort, Table S2: Selected features (in order of importance) by elastic net regularization for each dataset for assessment of subgroup HR+/HER2−, Table S3: Selected features (in order of importance) by elastic net regularization for each dataset for assessment of subgroup HR+/HER2−.
