# Supplementary material for: Multiparametric 18F-FDG PET/MRI-Based Radiomics for Prediction of Pathological Complete Response to Neoadjuvant Chemotherapy in Breast Cancer
Source: Cancers (Basel). 2022 Mar 29;14(7):1727. doi: 10.3390/cancers14071727 (PMC8996836; doi:10.3390/cancers14071727)
Supplement: Supplementary file 1 [file cancers-14-01727-s001.zip › cancers-1619941-supplementary.pdf]

Supplementary Material: Multiparametric 18F-FDG PET/MRI-Based Radiomics for Pre-diction of Pathological Complete Re-sponse to Neoadjuvant Chemotherapy in Breast Cancer

Lale Umutlu, Julian Kirchner, Nils-Martin Bruckmannn, Janna Morawitz, Gerald Antoch, Saskia Ting, Ann-Kathrin Bittner, Oliver Hoffmann, Lena Häberle, Eugen Ruckhäberle, Onofrio Antonio Catalano, Michal Chodyla, Johannes Grueneisen, Harald H. Quick, Wolfgang P. Fendler , Christoph Rischpler, Ken Herrmann, Peter Gibbs 11 and Katja Pinker

Table S1. Selected features (in order of importance) by elastic net regularization for each dataset for assessment of the entire cohort.

Data	1st Feature	2nd Feature	3rd Feature	4th Feature	5th Feature	6th Feature
ADC	mean absDev (FO)	gln (NGLDM)	run emphasis (RLM)	dcn (NGLDM)	energy (FO)	
T2	minimum (FO)	total energy (FO)	size zone variance (SZM)	joint maximum (GLCM)	inverse variance (GLCM)	gln (RLM)
Phase 1	energy (FO)	zln (SZM)	minimum (FO)	gln (NGLDM)		
Phase 2	gln (NGLDM)	energy (FO)	zln (SZM)	cluster prominence (GLCM)		
Phase 3	gln (NGLDM)	energy (FO)	size zone variance (SZM)			
Phase 4	energy (FO)	gln (NGLDM)	zln (SZM)			
Phase 5	energy (FO)	gln (NGLDM)	mean (FO)	zln (SZM)		
All Phases	energy (FO P4)	energy (FO P1)	gln (NGLDM P5)	minimum (FO P1)	size zone variance (SZM P3)	mean (FO P5)
All MR	energy (FO P4)	energy (FO P1)	gln (NGLDM P5)	minimum (FO T2)	mean absDev (FO ADC)	gln (NGLDM ADC)
PET	busyness (NGTDM)	ldhge (NGLDM)	zlnNorm (SZM)	contrast (NGTDM)	joint average (GLCM)	hde (NGLDM)
All MR and PET	busyness (NGTDM PET)	ldhge (NGLDM PET)	minimum (FO T2)	mean absDev (FO ADC)	minimum (FO P1)	2nd InfCorr (GLCM PET)

Table S2. Selected features (in order of importance) by elastic net regularization for each dataset for assessment of subgroup HR+/HER2−.

Data	1st Feature	2nd Feature	3rd Feature	4th Feature	5th Feature	6th Feature
ADC	dcnNorm (NGLDM)	zone percentage (SZM)	lde (NGLDM)	rlnNorm (RLM)	short run emphasis (RLM)	small zone emphasis (SZM)
T2	median (FO)	cluster shade (GLCM)	Haralick correlation (GLCM)	mean (FO)	szlgle (SZM)	root mean square (FO)
Phase 1	variance (FO)	standard deviation (FO)	maximum (FO)	range (FO)	mnAbsDev (FO)	root mean square (FO)
Phase 2	contrast (NGTDM)	short run emphasis (RLM)	gray level varaince (NGLDM)	invDiffMom (GLCM)	inverse difference (GLCM)	rlnNorm (RLM)
Phase 3	cluster shade (GLCM)	difference variance (GLCM)	10th percentile (FO)	coarseness (NGTDM)	interquartile range (FO)	Haralick correlation (GLCM)
Phase 4	dcnNorm (NGLDM)	invDiffNorm (GLCM)	invDiffMom (GLCM)	inverse difference (GLCM)	contrast (NGTDM)	dissimilarity (GLCM)
Phase 5	difference variance (GLCM)	minimum (FO)	cluster shade (GLCM)	coarseness (NGTDM)	2ndInfCorr (GLCM)	10th percentile (FO)
All Phases	minimum (FO P5)	minimum (FO P2)	cluster shade (GLCM P3)	10th percentile (FO P5)	difference variance (GLCM P5)	cluster shade (GLCM P2)
All MR	cluster shade (GLCM P3)	minimum (FO P5)	median (FO T2)	mean (FO P5)	10th percentile (FO P5)	cluster shade (GLCM T2)
PET	szhgle (SZM)	auto correlation (GLCM)	ldhge (NGLDM)	joint average (GLCM)	sum average (GLCM)	complexity (NGTDM)
All MR and PET	szhgle (SZM PET)	median (FO T2)	gray level variance (NGLDM P2)	auto correlation (GLCM_PET)	joint average (GLCM PET)	sum average (GLCM PET)

Table S3. Selected features (in order of importance) by elastic net regularization for each dataset for assessment of subgroup HR+/HER2−.

Data	1st Feature	2nd Feature	3rd Feature	4th Feature	5th Feature	6th Feature
ADC	entropy (NGLDM)	1st InfCorr (GLCM)				
T2	gln (NGLDM)	minimum (FO)	inverse variance (GLCM)	coefficient of dispersion (FO)	glnNorm (NGLDM)	
Phase 1	coefficient of dispersion (FO)	energy (FO)	lglze (SZM)	strength (NGTDM)	minimum (FO)	srlgle (RLM)
Phase 2	ldlge (NGLDM)	energy (FO)	energy (GLCM)	hglre (RLM)	zln (SZM)	
Phase 3	energy (FO)	szlgle (SZM)	szhgle (SZM)	energy (GLCM)		
Phase 4	zln (SZM)	glnNorm (NGLDM)	coefficient of dispersion (FO)	ldlge (NGLDM)		
Phase 5	coefficient of dispersion (FO)	energy (FO)	srlgle (RLM)			
All Phases	szlgle (SZM P3)	energy (FO P2)	zln (SZM P4)	energy (GLCM P2)	zln (SZM P2)	energy (GLCM P3)
All MR	szlgle (SZM P3)	energy (FO P2)	zln (SZM P4)	energy (GLCM P2)	zln (SZM P2)	energy (GLCM P3)
PET	2nd InfCorr (GLCM)	busyness (NGTDM)	strength (NGTDM)	entropy (NGLDM)		
All MR and PET	szlgle (SZM P3)	energy (FO P2)	zln (SZM P4)	energy (GLCM P2)	zln (SZM P2)	energy (GLCM P3)
